# Balancing work and health: A cross-sectional study on the relationship between paid employment and treatment adherence in Dutch people with a chronic condition

**DOI:** 10.1371/journal.pone.0342213

**Published:** 2026-10-13

**Authors:** Eva Aalbers, Hennie Boeije, Marcia Vervloet, Jany Rademakers, Liset van Dijk

**Affiliations:** 1 Nivel - Netherlands Institute for Health Services Research, Utrecht, The Netherlands; 2 Department of Family Medicine, School for Public Health and Primary Care (CAPHRI), Maastricht University, Maastricht, The Netherlands; 3 Department of Organisation Sciences, Vrije Universiteit Amsterdam, Amsterdam, The Netherlands; 4 Department of PharmacoTherapy, -Epidemiology & -Economics (PTEE), Groningen Research Institute of Pharmacy, Faculty of Science and Engineering, University of Groningen, Groningen, The Netherlands; Federal Teaching Hospital, Ido-Ekiti, NIGERIA

## Abstract

**Background:**

People with a chronic condition struggle with treatment adherence. At the same time, a majority of the working-age population with a chronic condition actively participates in the workforce. Having a paid job might affect the ability of people to incorporate treatments into their daily schedule, but it is currently unclear how and to which extent job-related factors affect treatment adherence.

**Methods:**

The Nivel Panel Stronger Together was used to collect self-reported data from 309 individuals with one or more chronic illnesses, who have paid work and use medication. Correlation analysis and univariate and multiple logistic regressions were performed to examine the effects of employment type, contract type, hours of work, extent of both physical and psychological demands, limitations at work, inclusion at work and work-related medication self-efficacy on treatment adherence. For this study, treatment adherence was defined as following the treatment plan as agreed with health professionals, and consisted of adherence to both medication and healthcare appointments.

**Results:**

The treatment adherence rate was 56%. Inclusion at work was positively associated with adherence (AOR 1.50, 95% CI 1.00–2.25, p = .049). Also, a relationship between moderate physical job demands and adherence was found (AOR 0.49, 95% CI 0.26–0.92, p = .026). No significant associations were found between adherence and the other studied variables.

**Conclusions:**

These results imply that the extent to which the working population with a chronic condition is able to adhere to their prescribed treatment, is not affected by practical workplace characteristics. Also, we found no evidence for an effect of self-efficacy, though results warrant further research. Still, working at a workplace where one feels acknowledged and valued improves the likelihood of being adherent, highlighting the importance of fostering an inclusive workforce for individuals with a chronic condition to improve adherence and workability.

## Introduction

People with a chronic condition are presented with a multitude of tasks and responsibilities to manage their health. One of those self-management tasks is adherence to treatment, which can be defined as ‘*following a treatment plan (care plan) agreed with the health professionals*” [1]. This includes both adhering to prescribed medication and arranging and attending appointments with healthcare professionals [2–4]. These tasks are a daily reality for a substantial part of the chronically ill population: over 90% of people with a chronic condition report the use of medication, 89% have regular contact with a general practice and 75% are under treatment of a medical specialist, with an average of 4.4 appointments per year [5]. Adherence to treatment is beneficial in achieving optimal treatment success [6,7]. Currently, medication adherence rates in people with a chronic condition are estimated to be around 50–75% [8,9], and the adherence rate to appointments ranges from 50 to 90% [10]. Research on the determinants of medication adherence shows that, among others, people struggle with the organization and practicalities of their medication treatment [8,9].

Presumably, a significant amount of the day-to-day management of medication and healthcare appointments occurs during working hours, or is otherwise affected by an individual’s work situation [11,12]. In the Netherlands, in 2024, 9,8 million people were participating in paid employment. Paid employment, as per the definitions of Statistics Netherlands, includes both those who have “*a contract with an economic unit to carry out work in return for financial remuneration*”, as well as those who are self-employed, both regardless of the working hours [13]. Approximately a quarter of the employed individuals report the presence of one or more chronic conditions [14], and 85% of employees with a chronic illness use medication at work [15]. Adherence to treatment is generally beneficial for employment, because it improves an individual’s health status and workability [11,16]. However, evidence on the effects of workforce participation on treatment adherence is inconclusive [10,17–22] and there is contradictory information on the specific factors which may explain this (lack of a) relationship. This knowledge gap also applies to the Dutch setting.

There are several mechanisms on how workforce participation could influence adherence. Following an ecological model approach and informed by the Theoretical Domains Framework [23], these factors can be situated on the individual, physical contextual and social level. First of all, on a contextual level, employment conditions such as hours of work and type of employment could affect treatment adherence. The time commitment required for healthcare appointments can conflict with work priorities [11,24], and combining work with the practicalities of medication taking behavior can be troublesome [25–27]. The regularity of a work schedule could enhance adherence by encouraging the formation and retention of new routines, while an interruption of such routines could impede adherence [12,28]. To illustrate: a study in Japan found that 23% of participants forgot to take their medication during the workday, especially when meals were skipped or meal-times were irregular [29]. Secondly, it is also possible that treatment adherence might be affected on a social level, through social relationships and organizational culture at the workplace. Although not extensively researched in a workplace setting, social support in general is found to be beneficial to adherence [30–32]. One approach to comprehend the social culture of an organization is by assessing the extent to which an organization is inclusive. An inclusive group values and accommodates individuality and personal autonomy, while simultaneously enabling a sense of belonging [33,34]. In such a group, treatment adherence can be supported and enabled.

Thirdly, on a more individual level, adherence at work could also be affected by a person’s self-efficacy: the belief that one is capable to adhere to one’s treatment, also under challenging circumstances [35]. There is a general consensus that self-efficacy enhances self-management behaviors [36] and adherence specifically [37–40], and thus it can be expected to also be the case at work.

Thus, to better understand the interplay between paid employment and treatment adherence, with this study we examine *to what extent working people with a chronic condition in the Netherlands are able to adhere to their prescribed medication and appointments, and how this is affected by work-related factors.* With this study we aim to provide more clarity on the relationship between work and adherence by clarifying and, where possible, reconciling inconsistent or inconclusive findings, and by identifying the factors underlying these associations. Based on the abovementioned rationales and the current literature, we expect that inclusion at work, work-related medication self-efficacy and being self-employed and will have a positive relationship with treatment adherence. At the same time, we expect that having a physically demanding job, a mentally demanding job or experiencing higher levels of limitations at work will have a negative relationship with treatment adherence. Finally, we also expect a relationship between hours of work and treatment adherence, and between the type of contract and adherence, although the nature of these relationship could go in either direction.

## Methods

### Participants

We performed a cross-sectional study among people aged 16–68 with a chronic physical condition in the Netherlands. Participants were drawn from the Nivel Panel Stronger Together (NPST; formerly the National Panel of the Chronically ill and Disabled), a nationwide panel focused on people with a chronic condition and/or a physical disability [41]. Within this panel, participants share their experiences and opinions in questionnaires on health and participation related topics, including work and self-management [5,42,43]. Panel members are recruited from random samples of general practices, drawn from the Dutch Database of General Practices [44]. Within each selected practice, a random one third of eligible patients with a chronic condition is invited to participate in the panel. Patients are eligible for participation if they have a somatic chronic disorder according to the International Classification of Primary Care (ICPC) [45], diagnosed by a certified general practitioner and are 16 years or older. Additionally, participants with physical disabilities are recruited from national population surveys conducted by Statistics Netherlands. People who are institutionalized, have a limited (<6 months) life expectancy or do not speak Dutch are excluded from participation. Eligible patients receive an invitation with an information letter and an informed consent form from their GP. Participants remain in the panel for a maximum of four years, new members are recruited annually.

To be included in this study, panel members needed to meet additional criteria, namely: being aged between 16 and 68 years old, having a paid job for at least 1 hour a week and being prescribed medication. The age group of the respondents (16–68) was chosen because of the minimum age for participating in the Nivel Panel Stronger Together on one hand (16) and the current retirement age in the Netherlands on the other hand (67 years and 3 months). To verify medication use, participants were asked if they currently use medication in the Spring 2020 and Spring 2021 questionnaires. Due to participant turnover between the questionnaires, this information was not available for 7.3% of the participants. However, 76.2% of them answered questions about medication adherence in the November 2020 questionnaire, similar to the average percentage of medication use in the target population. We therefore assumed that these participants use medication, and they were included in the study.

### Data collection

Questionnaires are distributed by the Dutch research institute Nivel twice a year (in autumn and spring) to the panel members either online or on paper, as per the participant’s preference. For the purpose of this study, the majority of data were collected from the NPST questionnaire of November 2020, complemented with self-efficacy data from the April 2022 questionnaire and medication use data from the April 2020 and April 2021 questionnaires. The data were compiled and accessed in May 2023.

### Measures

#### General information.

Sociodemographic information of participants included age, educational level (low, intermediate or high), sex, the number of chronic conditions (one, two or three or more) and the primary chronic condition. Participants missing information on single-item questions with no basis for imputation were excluded from the study. Missing items in multiple-item variables were imputed where appropriate, as described per measure below.

### Treatment adherence

Treatment adherence was inquired as part of the Partners in Health (PiH) scale, a validated scale that was developed to measure self-management skills of individuals with chronic illnesses [1,46]. This scale includes a two item sub-scale on treatment adherence: one question on using medication and carrying out treatment recommendations, and one question on arranging and attending (follow-up) appointments with healthcare professionals. Response options for both questions ranged from never to always on an 8-point scale, see S1 File. Both scores were combined to establish the treatment adherence score. Although this is a simplified operationalization of treatment adherence, due to the availability of data and the explorative nature of the study, we have used these two questions to serve as an indicator for treatment adherence. Due to the skewed distribution of adherence in this sample and the very low amounts of responses in the lowest adherence categories, the variable was dichotomized. When dichotomizing a self-reported adherence measure, it is recommended to do so at the 100% level, to account for the over-reporting that is often found in self-reported adherence rates [47]. Thus, participants who scored an 8 on both items, were classified as being fully adherent.

### Work participation

Participants were asked how many hours a week they worked. Furthermore, participants were asked about their employment contract type, with the options being self-employed, a permanent contract, a fixed term contract and/or a flexible contract, see S1 File.

### Work demands

Participants were asked two categorical questions regarding the extent to which they felt their job was physically and mentally demanding, with response options being not at all, slightly, moderately and heavily demanding, see S1 File.

### Inclusion at work

For participants working for an employer, the perceived organizational culture of their workplaces was measured with the perceived group inclusion scale (PGIS) [33]. This scale consists of 16 statements (α = 0.97 in our study) on membership, affection and authenticity, which had to be scored on a 5-point scale, ranging from 1 (strongly disagree) to 5 (strongly agree). The words ‘this group’ as used in the PGIS questionnaire were replaced with ‘the organization at which I work’, to fit the work setting, see S1 File. The total score was calculated as the average of all sub-questions, where a low score indicated a perception of inadequate inclusion at work. To limit loss of participants and statistical power, only participants missing 9 items or more (over 50% of the inclusion at work scale) (n = 7) were excluded. For those missing 8 items or less, missing data (n = 6) were replaced with the individual’s average score.

### Limitations at work

In order to measure limitations at work, a list of 11 items (α = 0.87 in our study) was devised on the extent to which participants had experienced issues or limitations at work as a consequence of their condition (e.g., movement at work, fatigue), see S1 File. These items could be rated with (1) no issues, (2) some, (3) many and (4) a lot of issues. The total limitation score was calculated by summing individual item scores, where a high score indicated a high perception of issues or limitations at work. This measure was treated as a continuous variable. Participants missing more than six items (n = 10) were excluded. For participants missing one to six items (n = 14), missing data were replaced with the individual’s median scale score.

### Work-related medication self-efficacy

In 2022, six questions on medication self-efficacy were included in the NPST questionnaire, where participants were asked to what extent they were confident in their ability to adhere to medication under several work-related circumstances (e.g., when their workday is busy, or when colleagues are around, see S1 File). Answering options were not sure, somewhat sure, very sure or not applicable. The work-related medication self-efficacy score was calculated by cumulating scores from the six questions (α = 0.74 in our study). For those missing one to three items (n = 3), missing data were replaced with the individual’s mean scale score. Participants missing four or more items were excluded. ‘Not applicable’ responses were recoded to ‘very sure’, assuming that these people did not experience particular difficulties. Participants marking all questions as ‘not applicable’ were excluded, assuming job loss or discontinued medication use between the 2020 and 2022 NPST questionnaires.

### Statistical analysis

Data were analyzed using Stata SE 16. Frequencies and percentages were calculated for all categorical variables, and means and standard deviations were calculated for numerical variables. Correlations with treatment adherence (yes/no) were calculated for all variables using the Chi-Squared (χ^2^) test for the categorical variables, and the Point-Biserial test for numerical variables.

The work-related variables type of employment, hours of work, extents of physical and psychological demands and limitations were used in univariate logistic regressions, where ‘full treatment adherence’ was the dichotomous dependent variable. Variables with a p-value of <.20 were then entered in multiple variable logistic regression analyses. Sex, age, number of conditions and level of education were included as control variables. Crude Odds Ratios (COR), Adjusted Odds Ratios (AOR) and 95% Confidence Intervals (CI) are displayed. For the subgroup of participants working for an employer, additional univariate and multiple variable logistic regressions were performed with type of contract and inclusion at work as independent variables. Each type of contract was considered a separate, binary variable. Furthermore, univariate and multiple logistic regressions were performed for participants who also completed the April 2022 questionnaire on medication self-efficacy. Here, work-related medication self-efficacy was seen as the independent variable. An alpha value of.05 was considered statistically significant.

### Sensitivity analysis

Sensitivity analyses were conducted to assess the robustness of three methodological choices. Firstly, the effect of setting a cut-off value of 8 for the outcome measure ‘full adherence’ was compared to thresholds of 7.5 or higher and 7 or higher to verify the possibility that our cut-off was to stringent and there was not so much over-reporting of adherence in our sample. Secondly, the implication of measuring ‘treatment adherence’ by grouping both adherence questions from the PiH scale was examined by separately analyzing each scale item with the independent variables to test if there were any variables that affected only appointment adherence or medication adherence. Finally, for the work-related medication self-efficacy score, the effect of defining the ‘not applicable’ replies as a perfect score (‘very sure’) was compared to treating these replies as missing values. We assumed that ‘not applicable’ responses indicated no particular difficulties. It is however possible that the participants had not encountered the described situations (such as taking medication when colleagues are around). For all sensitivity analyses, univariate and multivariate logistic regression analyses were performed as described above.

### Ethics approval and consent to participate

All data were collected and handled in accordance with the General Data Protection Act, the Netherlands Code of Conduct for Health Research, and the Netherlands Code of Conduct for Research Integrity. For the NPST, a program committee has been set up and a privacy regulation applies. In line with this privacy regulation, respondents received information on the panel and the use of data, and provided a written informed consent form. Data were pseudonymized, data linkage was performed based on this pseudonymized participant identifier and researchers did not have access to identifiable participant data. According to the Dutch Medical Research Involving Human Subjects Act, approval by a medical ethics committee is not mandatory for carrying out research in this panel. The program committee of the NPST has approved this study.

## Results

### Sample characteristics

A total of 2525 patients received a request to fill out the NPST questionnaire in November 2020, of whom 1969 (78%) participated; 361 participants met all inclusion criteria. A further 52 participants (14%) were eliminated due to insufficient completion of the single-item questionnaires, which led to a final sample of 309 participants. From this sample, some participants were included for further analysis on self-efficacy (n = 114) and for analysis on contract type and inclusion (n = 255). A flow-chart of the included participants can be found in Fig 1.

**Fig 1:**
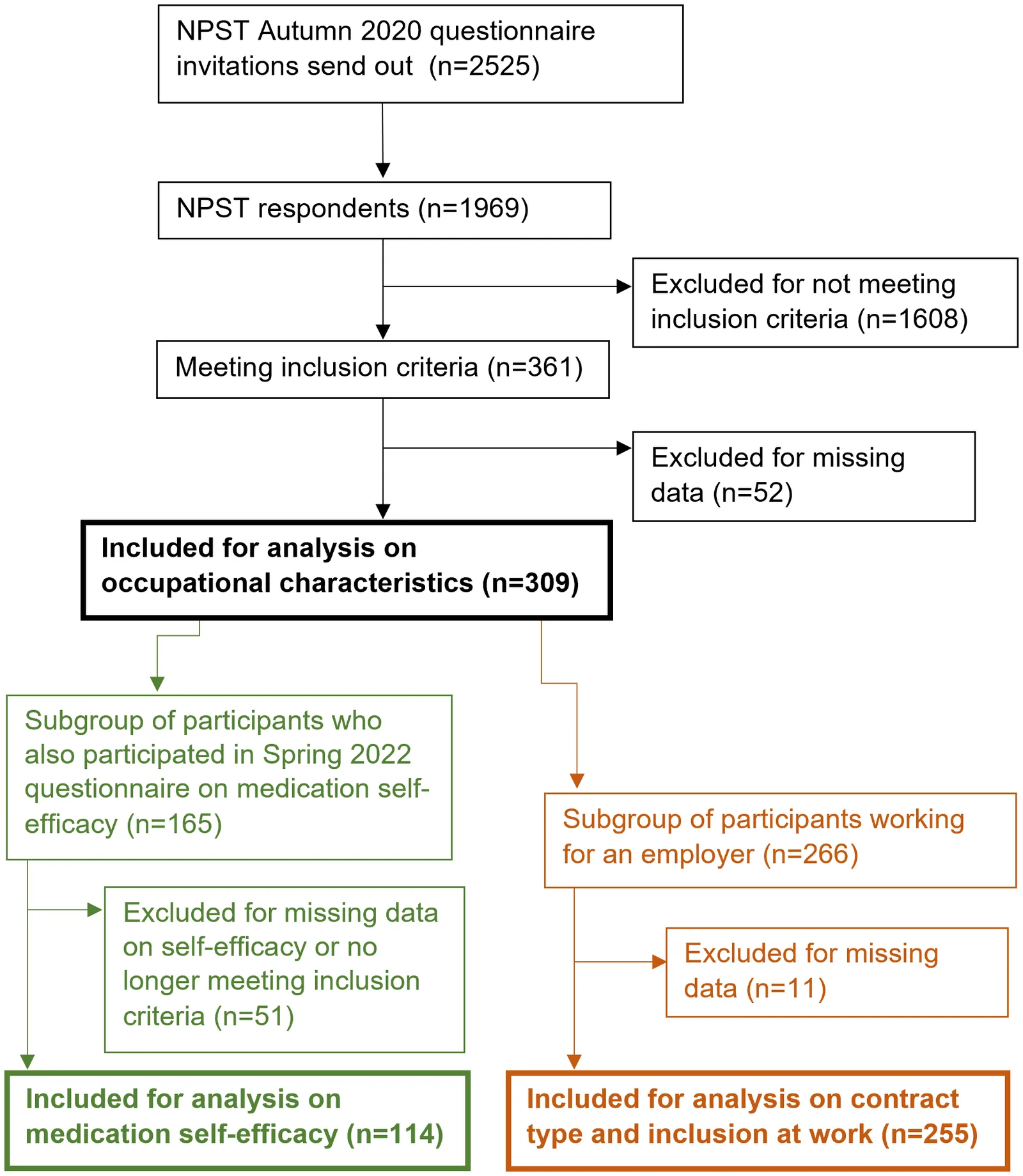
Flowchart participant selection.

Table 1 shows the demographic and workplace related characteristics of the included respondents.

**Table 1:** Sample characteristics of the study population (N = 309).

		n	%	M	SD
**Age**				53.8	10.7
**Sex**	Male	160	52%		
	Female	149	48%		
**Educational level**	Lower	35	11%		
	Intermediate	151	49%		
	Higher	123	40%		
**Number of chronic conditions**	One	179	58%		
	Two	91	29%		
	Three or more	39	13%		
**Type of employment**	As an employee	248	80%		
	Self-employed	43	14%		
	Both employee and self-employed	18	6%		
**Hours of work per week**				29.4	11.5
**Working fulltime**	(min. 36 hours per week)	124	40%		
**Extent of physical demands**	Not or hardly	98	32%		
	Light	73	24%		
	Moderate	96	31%		
	Heavy	42	14%		
**Extent of psychological demands**	Not or hardly	14	5%		
	Light	46	15%		
	Moderate	161	52%		
	Heavy	88	28%		
**Limitations at work**	(range: 11–37)			14.9	4.5
**Type of contract (n = 255)**	Permanent	219	86%		
*multiple answers possible	Fixed term	27	11%		
	Flexible	11	4%		
**Inclusion at work (n = 255)**	(range: 1–5)			3.7	0.7
**Medication self-efficacy at work (n = 114)**	(range 12–18)			17.4	1.4
**Average adherence score**	Treatment adherence			7.5	0.9
	Adherence to medication			7.4	1.2
	Adherence to appointments			7.5	1.0
**Dichotomized Adherence rates**	Fully adherent	174	56%		
	Fully adherent to medication	210	68%		
	Fully adherent to appointments	226	73%		

**Notes**: * p < .20 ** p < .05 ^a^ Adjusted for sex, age, educational level and number of diagnoses

The sample consisted of 160 males and 149 females, with an average age of 53.8 years old (SD 10.7). Most participants (58%) reported having one condition, 29% of the participants reported two conditions, and 13% of the participants reported 3 or more chronic health conditions. The most reported chronic conditions were cardiovascular diseases (19%) and asthma/CPOD (19%), followed by neurological conditions (9%), diabetes mellitus (9%), musculoskeletal conditions (7%), chronic digestive diseases (7%), cancer (6%) and other conditions (23%). Participants on average worked almost 30 hours per week, ranging from 1 to 65 hours, with a majority (60%) working part-time (< 36 hours per week). Most participants worked for an employer only (80%), a small portion worked fully self-employed (14%) and some did both (6%).

### Effects of work-related factors on treatment adherence

Participants on average rated their medication adherence with a 7.4 (SD 1.2) and their appointment adherence with a 7.5 (SD 1.0). Merging both types of adherence resulted in an average overall adherence score of 7.5 (SD 0.9). The majority of the participants scored a 7 or higher, with 14% scoring a 7, 17% scoring a 7.5 and 56% of the participants scoring 8, the maximum score, indicating full adherence. Overall, 68% of the participants said to always adhere to their prescribed medication, and 73% said to always follow up on their appointments. Medication adherence and appointment adherence were significantly associated to each other in our study, χ^2^ (1)= 31,51, p < .001.

Out of all the workplace characteristics (type of employment, hours of work, extents of physical and psychological demands, and limitations at work), only the level of physical demands at the workplace was significantly related to adherence, χ^2^ = 7.87, *p* = .049 (n = 309),. Participants experiencing moderate physical demands were less often fully adherent (45%), while participants who indicated no, light and heavy physical demands demonstrated higher levels of full adherence (62%, 59% and 64% respectively). Also after taking into account sex, age, educational level and number of diagnoses, the odds of being fully adherent were significantly lower for people who reported a moderate level of physical demands at their job (AOR = 0.49, 95% CI 0.26–0.92), compared to people with no physical demands, see Table 2.

**Table 2:** The effects of work-related variables on treatment adherence rates (n = 309).

		Univariate		Multivariate ^a^	
		COR (95% CI)	p-value	AOR (95% CI)	p-value
**Extent of physical demands**	Not or hardly	1.0		1.0	
	Lightly	0.87 (0.47-1.62)	.658	0.85 (0.44-1.63)	.620
	Moderately	0.49 (0.28-0.87)	.015**	0.49 (0.26-0.92)	.026**
	Heavily	1.09 (0.51-2.32)	.819	1.01 (0.44-2.32)	.981
**Extent of psychological demands**	Not or hardly	1.0		1.0	
	Lightly	0.30 (0.07-1.21)	.090*	0.34 (0.08-1.44)	.143
	Moderately	0.36 (0.10-1.35)	.131*	0.47 (0.12-1.87)	.287
	Heavily	0.31 (0.08-1.20)	.090*	0.41 (0.10-1.67)	.211
**Limitations at work**		1.01 (0.96-1.07)	.577	–	
**Type of employment**	Self-employed	0.82 (0.47-1.44)	.499	–	
	With employer	1.14 (0.60-2.18)	.688	–	
**Hours of work per week**		1.00 (0.98-1.02)	.701	–	
**Working fulltime**	0 = no, 1 = yes	1.07 (0.67-1.69)	.783	–	

**Notes**: * p < .20 ** p < .05 ^a^ Adjusted for sex, age, educational level and number of diagnoses

Data on contract type and inclusion at work were available for 255 of the participants working for an employer. In this group, those with a fixed term contract (n = 27) were less often fully adherent (37%) than those with other types of contracts (58%), χ^2^ = 4.50, *p* = .039 (n = 255). Nonetheless, the association of the type of contract did not hold in a multivariate regression, as shown in Table 3.

**Table 3:** The effects of contract type and inclusion at work on treatment adherence rates (n = 255).

		Univariate		Multivariate ^a^	
		COR (95% CI)	p-value	AOR (95% CI)	p-value
**Type of contract**	Permanent contract	1.69 (0.83-3.44)	.146*	0.56 (0.12-2.56)	.453
	Fixed term contract	0.43 (0.19-0.98)	.043*	0.21 (0.04-1.22)	.082
	Flexible contract	0.95 (0.28-3.21)	.938	–	
**Inclusion at work**		1.44 (0.98-2.10)	.063*	1.50 (1.00-2.25)	.049**

**Notes**: * p < .20 ** p < .05 ^a^ Adjusted for sex, age, educational level and number of diagnoses

On the contrary, the perceived level of inclusion did not show a significant relationship with adherence on its own (r_pb_ = 0.12, *p* = .06), but it did so after adjusting for personal characteristics in the multivariate analysis. The odds of being fully adherent increased as the participants felt more included at work (AOR = 1.50, 95% CI 1.00–2.25).

Self-efficacy data were available for a subsample of 114 participants. No significant association was found between work-related medication self-efficacy and adherence, as shown in Table 4.

**Table 4:** The effects of work-related medication self-efficacy on treatment adherence rates (n = 114).

		Univariate		Multivariate ^a^	
		COR (95% CI)	p-value	AOR (95% CI)	p-value
**Work-related medication self-efficacy**		1.29 (0.96-1.73)	.089*	1.41 (0.98-2.01)	.061

**Notes**: * p < .20 ^a^ Adjusted for sex, age, educational level and number of diagnoses

### Sensitivity analysis

Sensitivity analysis (see S2 File tables A1-A3) on the threshold for being classified as fully adherent showed that the level of physical demands lost significance after lowering the threshold to a 7+ and to a 7.5 + score. Inclusion at work was more significant in both scenarios, with AOR 1.92, 95% CI 1.13–3.28, p = .016 at a threshold of 7, and AOR, 2.26, 95% CI 1.44–3.56, p < .001 at a threshold of 7,5 + . The contract type ‘flexible contract’ only showed significance (AOR = 0.21, 95% CI 0.05–0.85, p = .028) at an adherence cut-off score of 7 + . Sensitivity analysis regarding combining medication and appointment adherence (see S2 File tables A4-A6) showed that the level of physical demands was significantly related to both appointment adherence and the combined score, but not to medication adherence alone. Inclusion was only significantly related to the combined score, although the odds ratios were nearing significance for both medication adherence (AOR 1.48, 95% CI 0.99–2.21, p = .059) and treatment adherence (ARO 1.50, 95% CI 0.97–2.30, p = .066). The other sensitivity tests did not show any substantial divergences.

## Discussion

With this study, we aimed to investigate the role that work-related factors play in adherence to treatment. We found a positive association between inclusion at work and adherence as well as a negative association between a moderate level of physical demands at work and adherence. None of the other factors were found to be significantly related to adherence in this study.

Firstly, we found that individuals who felt included at work were more likely to be fully adherent. To our knowledge, this interaction was not studied before. Nonetheless, there are several aspects of inclusion that may help to explain this relationship. Feeling valued and included might be an indication of low experience of stigma, and stigma at the workplace was found to lower adherence [21]. Additionally, medication and appointment adherence might require practical adjustments in the workplace, such as more flexibility in work hours, facilitating a safe space to take medication, etcetera. Employees might feel more valued when their employer is accommodating to their specific situation, and, vice versa, feeling included at work might encourage people to raise their voice in the organization, and voice their needed workplace adjustments [31,48,49]. Furthermore, in a supportive, accommodating workplace, people are more inclined to disclose their health status [50], which aids adherence [51].

Secondly, the amount of hours of work per week, the contract type, the level of self-employment and experienced limitations at work were not associated with adherence. Some other studies did find associations between adherence of occupational characteristics [29,52–55], but our findings add to the body of evidence where also no effects of contract type, job type, work schedule, overtime hours and self-employment were found [29,52,53,56]. This suggests that the practical organization of one’s job does not affect the extent to which they are able to adhere to their prescribed treatment.

It is also possible that the relationship between occupational characteristics and treatment adherence is more complex, and involves characteristics that were not included in this study. Possibly, the ability to combine work and treatment does not depend on the level of self-employment and on work hours, but more so on flexibility and autonomy within ones job [55,57]. It is also possible that certain occupational characteristics have opposing effects for different people. For example, self-employment could provide flexibility to schedule healthcare appointments for some individuals, whereas for others this could lead to increased job strain and excessive work hours, leaving little room for doctor appointments [55]. More in-depth research would be needed to verify these hypotheses.

Thirdly, the results of this study suggest that the level of adherence among working people with a chronic condition is not associated with their own self-efficacy. Although in general self-efficacy is beneficial for treatment adherence [38–40], we did not find any other studies on work-related medication self-efficacy. The high levels of work-related medication self-efficacy that were found in this study indicate that individuals are confident in their capabilities of adhering to their medication, even under challenging circumstances at work. Noteworthy, self-efficacy data was collected 1.5 years after collecting data on treatment adherence. During this time period, participants may have increased their self-efficacy, since self-efficacy is a dynamic construct that can change over time through acquiring new information and experiences [35,58]. Also, treatment adherence may have fluctuated and treatment regimens may have been altered. The time gap also reduced data availability due to participant attrition, leading to a smaller subsample. This might have contributed to the lack of relationship in this particular study. Moreover, the results narrowly missed the conventional level of statistical power (p = 0.061), and there are still indications of a strong trend towards clinical relevancy. Given the narrow range of the self-efficacy scale [12–18] and the high mean score (17.4 with a SD of 1.4), an AOR of 1.41 represents a major shift in adherence likelihood. The relatively small sample size (n = 114) and the ceiling effect in self-efficacy scores may have contributed to these results, and the lack of association may thus be an indication of limited statistical power rather than lack of potentially interesting association. Further research should be performed to collect more evidence on the role of self-efficacy on treatment adherence in the workplace setting.

Finally, our study found that individuals who reported a moderate level of physical demands at their job, were showing lower adherence rates than those with physically non-demanding jobs. Finding a lower adherence rate in people with moderate job demands is in line with previous research [54,59], especially in jobs with high decision latitude [57]. A high decision latitude – the flexibility and autonomy to make decisions on the organization of one’s job- might make it easier to incorporate treatment regimens into work. Possibly, the jobs in which people have more autonomy and decision latitude, such as management jobs and academic, knowledge-based functions fall in the low physical demand categories, which could explain the higher adherence in this group. Furthermore, people with higher adherence rates might do so because they experience a higher burden of disease in daily life [21], and thus also might opt for jobs with lower physical strain. Surprisingly, our study did not find a significant difference in adherence rates between no, low and highly physical demands. Although not statistically significant, we did find a higher level of adherence in the group of participants with highly physical jobs. We did not find other literature to explain this non-linearity. It is possible that people with a highly physically demanding job have a higher risk perception, and are more aware of the potential benefits of high adherence on their ability to work. Higer adherence might be crucial for them to continue performing their job duties. Higher perceived benefits of prescribed medication were found to have a positive effect on adherence rates [21]. Still, it is also possible that the pattern observed in our data was sample-specific. Replication is merited to verify the results and test the abovementioned hypotheses.

### Strengths and limitations

A strength of this study is that the internal validity of this study benefited from a relatively large sample size of over 300 doctor-diagnosed participants. Considering the rule of thumb to have a minimum sample size of 10 observations per independent variable, and the total of 27 independent variables in our study, we have also achieved sufficient statistical power [60]. Furthermore, this study is the first to study the interactions between work-related characteristics and treatment adherence within the Dutch context. Previous studies were mostly performed in other countries (such as Japan and Korea) with different cultural, social and occupational circumstances. This study is, to the best of our knowledge, also the first study to measure the level of medication self-efficacy with questions specifically tailored to fit the work setting, complementing the notion that self-efficacy is behavior and situation specific [35].

Nonetheless, several limitations should be considered when interpreting the findings, mostly due to the reliance on secondary data. Firstly, the use of self-reported adherence could have introduced bias, as participants may have under- or overestimated their actual adherence rates [47]. Additionally, adherence was measured with a two-item question only, for which a subscale was used that has not been validated as a stand-alone scale. The use of a two-question only instrument might have over-simplified treatment adherence, which is a complex and multifaceted construct, requiring a more comprehensive assessment. The use of a validated, adherence specific scale and objective measures, such as pill counts, electronic monitoring and healthcare usage data for appointment adherence, or multi-method measures, might provide a more accurate representation, for which studies by Vitolins et al. [61], Stirratt et al. [47], Forbes et al. [62] and Dunbar-Jacob and Zhao [63] provide valuable insights and suggestions. Also, although common in adherence research, the dichotomization of adherence may have decreased the sensitivity of the outcome measure, resulting in the loss of statistical power [64]. Although dichotomization helped mitigating the effects of our highly negatively skewed data, it also increased the risk of a Type II error. Possibly, the lack of relationships between adherence and some of the work-related factors in our study can be ascribed to the dichotomization of the data and the loss of detailed information. Nonetheless, the sensitivity analyses did not show major changes in outcomes. Although dichotomization may have masked small or subtle effects, this study was an explorative study to identify the most relevant work-related circumstances that aid optimal adherence. Where our study provides an initial understanding of the relationship between work and treatment adherence, a repeated study using a more sensitive and detailed adherence measure could build on our findings by detecting more subtle effects and shedding light on the specific aspects of adherence affected by workforce participation.

Secondly, the data collection during the Covid-years might have affected the temporal validity of this study. The pandemic introduced unprecedented challenges and changes in workforce dynamics, such as remote work, altered work hours, and heightened job insecurity, which could have potentially influenced both medication adherence and perceptions related to work. Thirdly, while this study delved into adherence in general, it did not specifically target medication use and appointment adherence within the workplace context, nor did it differentiate between various types of medication and medication regimens. Fourthly, combining data from multiple questionnaires based on availability, resulted in a time gap between measures, which could have compromised the validity of the analyses.

### Implications for practice and further research

The results of this study stress the importance of fostering an inclusive work environment. As inclusion was primarily operationalized as social inclusion (measuring belonging and authenticity), and assuming that our hypotheses concerning the possible mechanisms underlying the relationship between inclusion and adherence as discussed prior are valid, fostering inclusion is connected to a culture of openness and support. Employers, human resource professionals and colleagues should strive to promote a culture in which people with a chronic condition feel accepted and empowered. Although there is little empirical evidence regarding effective policies and strategies to enhance a culture of inclusion at the workplace, a review by Nguyen [34] suggests practices such as intentional learning, inclusive leadership, training and reverse mentoring. Notably, as our study was exploratory, further research should identify how inclusiveness should be operationalized to tangibly enhance treatment adherence. Additionally, the findings of this study should inspire health care professionals and occupational health specialists to not only take into account personality traits and psychological factors when it comes to treatment adherence, but also be aware of the positive impact of the social work-context in which the individual resides.

Next to this, the study indicates that the relationship between workplace related characteristics and adherence is complicated. As this study was a explorative study providing an initial insight in the relationship between work and adherence, there is a need for more in-depth, qualitative research to better understand the effects of specific workplace factors on an individual level and get more insight into personal experiences. Additionally, qualitative research could further explore the concept of inclusion at work and the roles of stigma, disclosure and workplace adaptations, as well as the impact of different types of medication and treatment regimens. In addition, more rigorous research is needed on several work-related concepts such as flexibility and autonomy at the workplace to better understand their interactions and impact on adherence. A repeated study on the effects of work-related medication self-efficacy would benefit from data collected at a single point in time with a more reliant, multi-method adherence measurement.

## Conclusions

Based on the results of this study, we conclude that, within the Dutch work context, adherence is mostly affected by the social context in which people with a chronic condition perform their jobs, and not so much depends on the practical organization of one’s job. We found no evidence for an effect of self-efficacy, but based on this study alone, self-efficacy cannot be discounted as a relevant factor for adherence in the context of work. Still, when individuals with chronic conditions feel valued, acknowledged, and integrated within their professional settings, they are enabled to adhere to their treatments and medication regimens.

## Supporting information

S1 File Survey questions collected from NPST questionnaires. (PDF)

S2 File Sensitivity analyses. (PDF)
