# Supplementary material for: Balancing work and health: A cross-sectional study on the relationship between paid employment and treatment adherence in Dutch people with a chronic condition
Source: PLoS One. 2026 Oct 13;21(10):e0342213. doi: 10.1371/journal.pone.0342213 (PMC13660926; doi:10.1371/journal.pone.0342213)
Supplement: Supplementary file 1 [file pone.0342213.s001.pdf]

Additional File 1 - Survey questions collected from NPCD questionnaires (translated from Dutch)

Medication use (April 202 and April 2021)

Have you, in the past 14 days, used medications that were prescribed by a doctor?

☐ No

☐ Yes

Treatment adherence
* Used instrument: The Partners in Health scale

I take medications and carry out treatments asked by my doctor or health worker

0	1	2	3	4	5	6	7	8
☐	☐	☐	☐	☐	☐	☐	☐	☐
Never				Sometimes				Always

I arrange and attend appointments as proposed by my doctor or health worker.

0	1	2	3	4	5	6	7	8
☐	☐	☐	☐	☐	☐	☐	☐	☐
Never				Sometimes				Always

Work participation

Do you currently perform paid work (as an employee or self-employed)
(*Multiple answers possible)*
☐ No
☐ Yes, …… hours a week as an employee
☐ Yes, …… hours a week self-employed

What type of employment do you currently have? (*Multiple answers possible)*

☐ Self-employed
☐ Permanent contract (full-time or part-time)
☐ Temporary contract with a fixed number of hours per week (full-time or part-time)
☐ Flexible contract (temporary agency work, on-call work, substitute work))

Work demands

Can you indicate to what extent your work is physically demanding?
☐ Highly physically demanding
☐ Moderately physically demanding
☐ Lightly physically demanding
☐ Not or hardly physically demanding

Can you indicate to what extent your work is mentally demanding?
☐Highly physically demanding
☐ Moderately physically demanding

☐ Lightly physically demanding
☐ Not or hardly physically demanding

Inclusion at work

*Used instrument: The Perceived Group Inclusion Scale, adapted to the work-setting

Could you indicate to what extent you agree with each statement?

**The organization I work for…..**	**Strongly disagree**	**Disagree**	**Neutral**	**Agree**	**Strongly agree**
…likes me	☐	☐	☐	☐	☐
…appreciates me	☐	☐	☐	☐	☐
…is pleased with me	☐	☐	☐	☐	☐
…cares about me	☐	☐	☐	☐	☐

**The organization I work for…..**	**Strongly disagree**	**Disagree**	**Neutral**	**Agree**	**Strongly agree**
…gives me the feeling that I belong	☐	☐	☐	☐	☐
…gives me the feeling that I am part of this group	☐	☐	☐	☐	☐
…gives me the feeling that I fit in	☐	☐	☐	☐	☐
…treats me as an insider	☐	☐	☐	☐	☐

**The organization I work for…..**	**Strongly disagree**	**Disagree**	**Neutral**	**Agree**	**Strongly agree**
…allows me to be authentic	☐	☐	☐	☐	☐
…allows me to be who I am	☐	☐	☐	☐	☐
…allows me to express my authentic self	☐	☐	☐	☐	☐
…allows me to present myself the way I am	☐	☐	☐	☐	☐

The organization I work for…..	Strongly disagree	Disagree	Neutral	Agree	Strongly agree
…encourages me to be authentic	☐	☐	☐	☐	☐
…encourages me to be who I am	☐	☐	☐	☐	☐
…encourages me to express my authentic self	☐	☐	☐	☐	☐
…encourages me to present myself the way I am	☐	☐	☐	☐	☐

Limitations at work

Can you indicate to what extent you experience issues or limitations at work, as a consequence of your health? *(please answer all questions)*

I experience issues/limitations at work as a consequence of my health when it comes to….

….	None	Some	Many	Very Many
… commuting to and from work	☐	☐	☐	☐
… movement within the workplace	☐	☐	☐	☐
… reading	☐	☐	☐	☐
… working precisely	☐	☐	☐	☐
… comprehension of speech	☐	☐	☐	☐
… fatigue	☐	☐	☐	☐
… stress	☐	☐	☐	☐
… performing and finishing work	☐	☐	☐	☐
… focus	☐	☐	☐	☐
… pain	☐	☐	☐	☐
… shame or guilt	☐	☐	☐	☐

Others, namely:

……

……

Work-related medication self-efficacy

Below are 6 items on how confident you are at a certain moment or in a certain situation, that you use your medication well. U can always choose from four answer options.

**To what extend are you confident that you use your medication well….**	Not sure	Some what sure	Very sure	Not applicable
When you are working.	☐	☐	☐	☐
When your workday is busy.	☐	☐	☐	☐
When colleagues are around.	☐	☐	☐	☐

When you are not sure if, after using medication, you are able to get to work.	☐	☐	☐	☐
When you are not sure if, after using medication, you are able to continue working.	☐	☐	☐	☐
When, because of work, you cannot use your medication at the desired time.	☐	☐	☐	☐
