# Supplementary material for: Balancing work and health: A cross-sectional study on the relationship between paid employment and treatment adherence in Dutch people with a chronic condition
Source: PLoS One. 2026 Oct 13;21(10):e0342213. doi: 10.1371/journal.pone.0342213 (PMC13660926; doi:10.1371/journal.pone.0342213)
Supplement: Supplementary file 2 [file pone.0342213.s002.pdf]

Additional File 2 - Sensitivity analyses

Table A1 – Effects of adjusting the threshold for full adherence on the relationship between work-related variables and treatment adherence (N=309)

		Threshold of 7				Threshold of 7.5			
		**Univariate**		**Multivariate [a]**		**Univariate**		**Multivariate [a]**	
		**COR (95% CI)**	**p-value**	**AOR (95% CI)**	**p-value**	**COR (95% CI)**	**p-value**	**AOR (95% CI)**	**p-value**
**Extent of physical demands**	Not or hardly				-			-	-
	Lightly	0.81 (0.31-2.10)	.662	0.82 (0.31-2.17)	.687	1.22 (0.60-2.50)	.587	-	-
	Moderately	0.57 (0.24-1.32)	.190*	0.57 (0.23-1.40)	.218	0.68 (0.38-1.28)	.233	-	-
	Heavily	0.68 (0.23-2.02)	.489	0.62 (0.20-1.99)	.427	0.97 (0.42-2.20)	.933	-	-
**Extent of psychological demands**	Not or hardly			-	-				
	Lightly	0.32 (0.04-2.74)	.296	-	-	0.18 (0.02-1.48)	.109*	0.16 (0.02-1.39)	.098
	Moderately	0.57 (0.07-4.65)	.604	-	-	0.20 (0.03-1.61)	.132*	0.19 (0.02-1.54)	.120
	Heavily	0.49 (0.06-4.07)	.507	-	-	0.19 (0.02-1.56)	.123*	0.18 (0.02-1.48)	.110
**Limitations at work**		0.98 (0.92-1.05)	.651	-	-	0.99 (0.94-1.05)	.764	-	-
**Type of employment**	Self-employed	0.63 (0.29-1.33)	.224	-	-	0.71 (0.39-1.31)	.274	-	-
	With employer	1.33 (0.55-3.22)	.532	-	-	1.35 (0.68-2.71)	.395	-	-
**Hours of work per week**		0.99 (0.97-1.02)	.658	-	-	0.99 (0.97-1.02)	.563	-	-
**Working fulltime**	0=no, 1=yes	0.67 (0.34-1.29)	.227	-	-	0.86 (0.52-1.42)	.550	-	-

Notes: * $p < .20$ [a] Adjusted for sex, age, educational level and number of diagnoses; COR= Crude Odds Ratio, AOR= Adjusted Odds Ratio, CI = Confidence Interval

Table A2 – Effects of adjusting the threshold for full adherence on the relationship between contract type and inclusion at work and treatment adherence (N=255)

		Threshold of 7				Threshold of 7.5			
		**Univariate**		**Multivariate [a]**		**Univariate**		**Multivariate [a]**	
		**COR (95% CI)**	**p-value**	**AOR (95% CI)**	**p-value**	**COR (95% CI)**	**p-value**	**AOR (95% CI)**	**p-value**
	Permanent contract	1.79 (0.71-4.50)	.215			1.65 (0.78-3.46)	.190*	1.87 (0.81-4.30)	.141
**Type of contract**	Fixed term contract	0.84 (0.27-2.60)	.759			0.59 (0.26-1.37)	.221		
	Flexible contract	0.24 (0.07-0.86)	.028**	0.21 (0.05-0.85)	.028**	0.64 (0.18-2.24)	.481		
**Inclusion at work**		1.86 (1.10-3.12)	.019**	1.92 (1.13-3.28)	.016**	2.13 (1.37-3.29)	.001**	2.26 (1.44-3.56)	<.001**

Notes: * p < .20 ** p< .05 [a] Adjusted for sex, age, educational level and number of diagnoses; COR= Crude Odds Ratio, AOR= Adjusted Odds Ratio, CI = Confidence Interval

Table A3 – Effects of adjusting the threshold for full adherence on the relationship between adherence and work-related mediation self-efficacy (N=114)

	Threshold of 7				Threshold of 7.5			
	**Univariate**		**Multivariate [a]**		**Univariate**		**Multivariate [a]**	
	**COR (95% CI)**	**p-value**	**AOR (95% CI)**	**p-value**	**COR (95% CI)**	**p-value**	**AOR (95% CI)**	**p-value**
**Work-related medication self-efficacy**	1.25 (0.89-1.76)	0.204	1.52 (0.96-2.36)	.065	1.17 (0.89-1.57)	.261	-	-

Notes: [a] Adjusted for sex, age, educational level and number of diagnoses; COR= Crude Odds Ratio, AOR= Adjusted Odds Ratio, CI = Confidence Interval

Table A4 – Effects of separately analyzing the effects of work-related factors on medication adherence and appointment adherence (N=309)

		Medication adherence				Appointment adherence			
		**Univariate**		**Multivariate [a]**		**Univariate**		**Multivariate [a]**	
		**COR (95% CI)**	**p-value**	**AOR (95% CI)**	**p-value**	**COR (95% CI)**	**p-value**	**AOR (95% CI)**	**p-value**
**Extent of physical demands**	Not or hardly								
	Lightly	0.91 (0.47-1.76)	.788	-	-	1.15 (0.54-2.45)	.718	1.13 (0.52-2.49)	.752
	Moderately	0.73 (0.40-1.34)	.310	-	-	0.43 (0.23-0.82)	.010**	0.43 (0.22-0.87)	.020**
	Heavily	1.05 (0.47-2.33)	.903	-	-	0.77 (0.33-1.78)	.539	0.66 (0.26-1.65)	.369
**Extent of psychological demands**	Not or hardly								
	Lightly	0.16 (0.02-1.33)	.090*	0.15 (0.02-1.29)	.084	0.28 (0.06-1.43)	.126*	0.33 (0.06-1.74)	.191
	Moderately	0.17 (0.02-1.30)	.088*	0.16 (0.02-1.32)	.089	0.46 (0.10-2.13)	.319	0.61 (0.12-3.03)	.548
	Heavily	0.13 (0.02-1.08)	.059*	0.13 (0.01-1.05)	.055	0.53 (0.11-2.57)	.432	0.74 (0.14-3.82)	.716
**Limitations at work**		1.01 (0.96-1.07)	.636	-	-	0.98 (0.93-1.04)	.542	-	-
**Type of employment**	Self-employed	1.05 (0.58-1.93)	.868	-	-	0.77 (0.42-1.42)	.400	-	-
	With employer	1.03 (0.52-2.05)	.937	-	-	1.38 (0.69-2.76)	.365	-	-
**Hours of work per week**		1.00 (0.98-1.02)	.905	-	-	1.00 (0.98-1.03)	.690	-	-
**Working fulltime**	0=no, 1=yes	1.26 (0.77-2.07)	.354	-	-	1.09 (0.65-1.83)	.732	-	-

Notes: * p < .20 ** p< .05 [a] Adjusted for sex, age, educational level and number of diagnoses; COR= Crude Odds Ratio, AOR= Adjusted Odds Ratio, CI = Confidence Interval

Table A5 – Effects of separately analyzing the effects of contract type and inclusion at work on medication adherence and appointment adherence (N=255)

		Medication adherence				Appointment adherence			
		**Univariate**		**Multivariate [a]**		**Univariate**		**Multivariate [a]**	
		**COR (95% CI)**	**p-value**	**AOR (95% CI)**	**p-value**	**COR (95% CI)**	**p-value**	**AOR (95% CI)**	**p-value**
**Type of contract**	Permanent contract	1.18 (0.56-2.46)	.663	-	-	1.99 (0.95-4.17)	.067*	0.76 (0.14-4.13)	.749
	Fixed term contract	0.69 (0.30-1.55)	.364	-	-	0.40 (0.18-0.90)	.027**	0.30 (0.05-1.96)	.209
	Flexible contract	0.85 (0.24-3.00)	.805	-	-	1.63 (0.34-7.76)	.537	-	-
**Inclusion at work**		1.46 (0.98-2.17)	.060*	1.48 (0.99-2.21)	.059	1.42 (0.94-2.15)	.099*	1.50 (0.97-2.30)	.066

Notes: * p < .20 ** p< .05 [a] Adjusted for sex, age, educational level and number of diagnoses; COR= Crude Odds Ratio, AOR= Adjusted Odds Ratio, CI = Confidence Interval

Table A6 – Effects of separately analyzing the effects of work-related medication self-efficacy on medication adherence and appointment adherence (N=114)

	Medication adherence				Appointment adherence			
	**Univariate**		**Multivariate [a]**		**Univariate**		**Multivariate [a]**	
	**COR (95% CI)**	**p-value**	**AOR (95% CI)**	**p-value**	**COR (95% CI)**	**p-value**	**AOR (95% CI)**	**p-value**
**Work-related medication self-efficacy**	1.07 (0.81-1.41)	.619	-	-	n.a.	n.a.	n.a.	n.a.

Notes: [a] Adjusted for sex, age, educational level and number of diagnoses n.a. = not applicable; COR= Crude Odds Ratio, AOR= Adjusted Odds Ratio, CI = Confidence Interval
